# The Impact of Periodontitis on Cardiovascular Disease: Mechanisms, Evidence, and Therapeutic Implications

**DOI:** 10.1155/ijod/3694736

**Published:** 2025-08-23

**Authors:** Zhongxiu Wang, Jiaqi Bao, Yuting Yang, Yingming Wei, Chen Li, Yaping Pan, Lili Chen

**Affiliations:** ^1^Department of Periodontology, The Second Affiliated Hospital, College of Medicine, Zhejiang University, Hangzhou 310009, China; ^2^Department of Periodontology, School and Hospital of Stomatology, China Medical University, Shenyang 110002, China

**Keywords:** cardiovascular disease, noncommunicable diseases, periodontitis, risk factor

## Abstract

**Objective:** Periodontitis, a highly prevalent chronic inflammatory disease caused by bacteria. Cardiovascular disease (CVD) is responsible for more than 17 million deaths globally each year. Both periodontitis and CVD are global noncommunicable diseases that share common risk factors. This review aimed to provide a guide for dentists and physicians, and improved treatment regimens for patients with both periodontitis and CVDs.

**Method:** This present review reports existing evidence in the literature to summarize the correlation between periodontitis and six types of CVD, discusses the existing epidemiological evidence, intermechanism connections, and the impact of periodontal therapy on cardiovascular health.

**Results:** Current research has emphasized the potential importance of periodontitis as a risk factor for CVD and has revealed various mechanisms of interaction between the two conditions. These mechanisms include oxidative stress, immune-inflammatory responses, and dysbiosis of the oral microbiota. Periodontitis may directly or indirectly induce systemic inflammation and oxidative stress by altering the circulation of oral microbiota, thereby affecting the occurrence and development of CVD.

**Conclusion:** By strengthening prevention strategies of periodontitis, we have the potential to prevent or ameliorate cardiovascular conditions. The review provides new perspectives and an indication of future directions for the prevention of CVD.

## 1. Introduction

Periodontitis, characterized by the destruction of the alveolar bone and loss of teeth, is a chronic inflammatory oral disease caused by bacteria. Periodontitis has a high prevalence and is the sixth most common disease globally. Increasing evidence suggests that severe periodontitis increases the risk of systemic disease. Based on this, the impact of periodontal infection on systemic conditions is termed “periodontal medicine” [1].

Cardiovascular disease (CVD) is a general term used to describe disorders of the heart and blood vessels. Heart disorders begin with atherosclerosis and develop into myocardial infarction, arrhythmia, heart failure (HF), cardiomyopathy, and heart valve disorders. As for blood vessel disorders, atherosclerosis may cause coronary heart disease (CHD) [2]. CVD has become the leading cause of death in Asian countries, and is responsible for 17.9 million deaths worldwide. Chronic infectious and inflammatory diseases are reportedly consistent with a higher risk of adverse CVD events [3].

**Figure 1:**
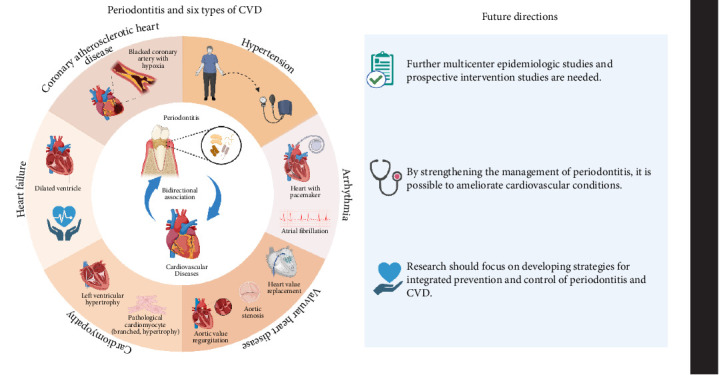
The impact of periodontitis on cardiovascular disease: mechanisms, evidence, and therapeutic implications.

Both periodontitis and CVD are global noncommunicable diseases that share common risk factors. Since the 1900s, an association between dental bacteremia and CVD has been reported [4]. An increasing number of studies have shown that periodontitis contributes to the development of CVD. However, the exact causal relationship between periodontitis and CVD remains unclear. Therefore, this review summarizes the correlation between periodontitis and six types of CVD, discusses the existing epidemiological evidence, intermechanism connections, and the impact of periodontal therapy on cardiovascular health (Figure 1). We aim to provide a resource for dentists and physicians, and have made recommendations for patients with both periodontitis and CVDs.

## 2. The Association Between Periodontitis and CHD

Coronary atherosclerotic heart disease, which is abbreviated to CHD, and is also known as ischemic heart disease, is the biggest single cause of mortality globally. Plaque deposition results in arterial narrowing, which is responsible for myocardial ischemia and myocardial infarction [5]. CHD shares similar characteristics with periodontitis, such as susceptibility of the elderly, smokers, and patients with lower educational achievement and lower socioeconomic status [4].

In 1988, a case–control study revealed significant differences in the community periodontal index with myocardial infarction attacks, indicating an association between oral health and CHD [6]. Further case–control studies have revealed that increased periodontal probing depth, furcation lesions, and bleeding on probing are associated with myocardial infarction [7]. Cross-sectional studies from many countries have also reported that patients with periodontitis exhibit a high incidence of CHD [8, 9]. Recently, a cohort study analyzed the risk of CHD in patients with periodontitis and reported that periodontitis was associated with an increased risk of CHD in middle-aged and elderly Chinese people [10]. One meta-analysis adjusted the confounding factors of various research studies and confirmed that the prevalence of CHD was significantly increased in periodontitis, proposing that periodontal disease is an independent risk factor for CHD [11]. Moreover, patients with periodontitis demonstrated enhanced carotid intima-media thickness, flow-mediated dilation, and arterial stiffness. Nepomuceno et al. [12] performed a meta-analysis of 19 studies and found that participants with chronic periodontitis presented significantly higher serum levels of low-density lipoprotein and triglycerides, but lower levels of high-density lipoprotein.

However, the pathogenic mechanisms underlying CHD remain unclear. One possible association is that severe periodontitis may lead to bacteremia, transporting the bacteria to coronary artery plaques [13]. *Porphyromonas gingivalis* (*P. gingivalis*), *Actinobacillus actinomycetemcomitans* (*A. actinomycetemcomitans*), and *Campylobacter rectus* (*C. rectus*) were the main pathogens of subgingival plaques that contribute to the development of periodontitis. They are also found in the atherosclerotic plaques of patients with chronic periodontitis [13]. Chukkapalli et al. [14] infected the gingiva of ApoE-null mice with a bacterial complex (including *P. gingivalis*, *Treponema denticola*, *Tannerealla forsythia*, and *Fusobacterium nucleatum*). *P. gingivalis* was observed in the aortic wall at 12 weeks, and *Treponema denticola* and *Fusobacterium nucleatum* were detected in the heart and aorta at 24 weeks. After 24 weeks of bacterial infection, the mice exhibited larger plaque areas and intimal/medial layer thickness ratios than those in the control group [14].

Periodontal pathogens may not only influence plaque formation but also affect the vessel endothelium. *P gingivalis* can invade into endothelial cells, and activate the toll-like receptors/nuclear factor kappa B pathway, suppressing BMAL1 transcription, releasing CLOCK, and finally elevating oxidative stress and inflammatory response in human aortic endothelial cells [15]. Gingipains from *P. gingivalis* and free soluble bacterial components of *A. actinomycetemcomitans* also promoted proatherogenic responses in endothelial cells [16].

Foam cells are monocytes or histiocytes that absorb lipids. These are characteristic pathological cells that appear in atherosclerotic plaques and play important roles in CHD. *P. gingivalis* induces foam cell formation from macrophages via outer membrane vesicles, lipopolysaccharide, and lysosomal integral membrane protein 2 [17]. In terms of the mechanism, *P. gingivalis* and its components upregulate the intake of oxidized low-density lipoprotein by activating nuclear factor kappa, extracellular signal-regulated kinase 1/2 and p65 pathways in macrophages [18].

Severe periodontitis accompanied by dysbiosis can increase systemic inflammation. Inflammation triggered by periodontal pathogens may affect immune responses. *P. gingivalis* infection can break the balance between regulatory T cells and T helper cells, aggravating the formation of foam cells and arterial plaques. The metabolism products also have a decisive position. Lipid metabolism (e.g., glycerophospholipid and sphingolipid) were found upregulated in patients with carotid atherosclerosis [19].

## 3. The Association Between Periodontitis and Hypertension (HTN)

HTN, characterized by a persistently elevated systolic blood pressure (SBP) or diastolic blood pressure (DBP), is a multifactorial syndrome often accompanied by a range of cardiovascular risk factors. Research exploring the association between periodontitis and HTN dates back to 1998, when Ogawa et al. [20] conducted an epidemiological study that revealed higher community periodontal indices in patients with HTN. Since then, several cross-sectional studies have examined the association between HTN and periodontitis. Muñoz Aguilera et al. [21] conducted a systematic review and meta-analysis to assess the prevalence of HTN among patients with periodontitis. They discovered that patients with moderate-to-severe periodontitis had a 20%–50% increased risk of HTN [21]. A recent meta-analysis by Lu et al. [22] found that individuals with periodontitis were significantly more likely to develop atherosclerotic cardiovascular disease in the presence of HTN. Additionally, patients with periodontitis exhibited elevated SBP and DBP by 4.49 mmHg and 2.03 mmHg, respectively, compared to periodontally healthy individuals [21]. A comprehensive analysis of data from the Oral Infections and Vascular Disease Epidemiology Study (INVEST), encompassing 653 patients from diverse ethnic backgrounds, delved into the intricate relationship between subgingival periodontal bacterial load and blood pressure [23]. Notably, individuals in the highest tertile of bacterial load exhibited nearly four times the likelihood of HTN risk compared to those in the lowest tertile, with males demonstrating a stronger correlation [23].

A randomized intervention trial assigned 101 patients with HTN and periodontitis to either an intensive periodontal therapy (sub- and supragingival scaling/chlorhexidine) or control periodontal therapy (limited to supragingival scaling) group. Remarkably, after 2 months, the intensive periodontal therapy group showed significant reductions in SBP (~11.1 mmHg) compared with the control periodontal therapy group [24]. These findings suggest that periodontal treatment may play a crucial role in the management of HTN, potentially leading to improved blood pressure control and enhanced sensitivity to antihypertensive medications in patients with good periodontal health.

In the pathogenesis of periodontitis, microbial plaques trigger inflammatory responses in the periodontal tissues. Neutrophils migrate from the bloodstream to the infection site, leading to elevated levels of pro-inflammatory mediators, thereby maintaining chronic inflammation. A study conducted by Tonetti et al. [25] explored the effects of periodontal treatment on endothelial function in patients with severe periodontitis. At the 6-month follow-up, flow-mediated dilatation demonstrated significant improvement, along with improvements in periodontal variables [25]. Periodontitis may serve as a source of inflammation and oxidative stress. In the long term, this can lead to functional and anatomical vascular changes, resulting in arterial stiffness, increased vascular resistance, and volume overload, ultimately contributing to elevated blood pressure.

Studies have emphasized the pivotal role of T cells in HTN. Activated T cells accumulate in perivascular tissues and release cytokines, including TNF-α, interleukin-6 (IL-6), and IL-17, which promote HTN development. IL-17 specifically mediates an increased inflammatory response, leading to the scavenging of nitric oxide (NO) from vessel walls [26]. This impairs NO-mediated vasodilation and promotes the recruitment of inflammatory cells to perivascular tissues [27]. Interestingly, dysregulated expansion of Th17 cells also plays a central role in periodontitis development. Individuals naturally lacking Th17 cells appear to be less susceptible to development of periodontitis [28]. Additionally, neutrophil dysfunction, oral–gut microbiota dysbiosis, and sympathetic nervous system overactivity are potential mechanisms involved in periodontitis and HTN. For instance, an epidemiological study on oral infections and vascular diseases revealed a direct relationship between subgingival periodontal bacterial levels and both SBP and DBP, as well as prevalence of HTN [23].

In summary, periodontitis and HTN exhibit intricate relationships. Evidence strongly suggests an association between periodontal health and blood pressure regulation. Addressing periodontal health may have significant implications in the prevention and management of HTN, offering a novel biological basis for future research and intervention strategies.

## 4. The Association Between Periodontitis and Arrhythmia

Atrial fibrillation (AF) is a common type of rapid arrhythmia and its incidence increases exponentially with age. A cross-sectional study including 5634 participants with complete data on their periodontal and AF status reported that the severity of the dental plaque index was strongly associated with the prevalence of AF after adjusting for several confounders (age, sex, high-sensitivity C-reactive protein [CRP], IL-6, body mass index, diabetes, smoking, and educational level) [29]. Zhang et al. [30] analyzed eight clinical trials with 4,328,355 patients and found that periodontitis was associated with new-onset AF in a dose-dependent manner and increased the risk of long-term arrhythmia in patients with AF. Miyauchi et al. [31] followed-up 596 patients with AF for 17.1 ± 14.5 months after radiofrequency catheter ablation to analyze late recurrence. Serum IgG titers against *P. gingivalis* (type IV) were associated with late recurrence (OR = 1.937, *p*=0.002) and could predict late AF recurrence (paroxysmal AF: HR = 1.569, *p*=0.04; nonparoxysmal AF: HR = 1.909, *p*=0.004) [31]. A cohort study conducted over 17 years found that severe periodontitis was associated with AF (HR = 1.31, 95% CI = 1.06–1.62). Participants who received regular dental care had a lower risk of AF than those who received episodic dental care (HR = 0.88, 95% CI = 0.78–0.99) [32]. Similarly, bacterial elimination strategies such as tooth brushing can reduce the incidence of AF [30].

Anatomically, the type (paroxysmal or nonparoxysmal) and duration of AF are related to the degree of atrial fibrosis. Clinical markers of periodontitis (e.g., bleeding on probing and periodontal probing depth) are positively correlated with left atrial appendage fibrosis. Periodontal inflamed surface area is also strongly correlated with AF (*R* = 0.57, *p*  < 0.0001; *β* = 0.016, *p*=0.0002), implying that periodontitis participates in the modification of atrial substrate [33].

Systemic inflammation is also associated with AF. Serum concentration of IL-6 was considerably higher in patients with persistent and paroxysmal AF than in healthy individuals. Consistent with this, IL-6 trans-signaling activation was also recapitulated in a mouse model of AF. Selective blockade of IL-6 trans-signaling by Sgp130Fc significantly reduces immune cell infiltration and oxidative stress in the mouse model of AF [34]. Periodontitis is an inflammatory disease which upregulates serum IL-6 levels depending on its severity. Immune cell infiltration analysis revealed that neutrophils and T cells play important roles in the pathogenesis of both periodontitis and AF [35]. Collectively, periodontitis contributes to the incidence of AF, possibly by upregulating the circulating levels of IL-6 and immune responses.

Predisposing genes are also associated with the incidence of AF and periodontitis. Bioinformatic analysis identified 21 common genes in periodontitis and AF using the Gene Expression Omnibus database. G protein signaling 1 (RGS1) was the optimal target gene. RGS1 is positively correlated with activated memory CD4 T cells and gamma-delta T cells but negatively correlated with CD8 T cells and regulatory T cells [35]. Moreover, RGS1 was positively correlated with classical pro-inflammatory cytokines, IL-1β and IL-6.

## 5. The Association Between Periodontitis and HF

Patients with HF had the worst CVD outcomes. Walther et al. [36] performed transthoracic echocardiography and periodontal screening to determine the association between periodontitis and HF. Severe periodontitis was significantly associated with mid-range HF and reduced ejection fraction. A cross-sectional study further reported that the incidence of HF in participants with moderate/severe periodontitis was 5.72 times higher than that of a no/mild periodontitis group (95% CI: 3.76–8.72, *p*  < 0.001) [37]. An increase in the number of missing teeth is associated with a higher risk of HF [38]. As for patients with HF combined with periodontitis, individuals who brushed their teeth ≥ 2 times/day (HR = 0.90, 95% CI = 0.82–0.98) or received professional dental cleaning ≥ 1 time/year (HR = 0.93, 95% CI = 0.87–0.99) showed decreased HF risk [38].

With the development of artificial intelligence, the machine learning model of the *K*-nearest neighbor algorithm can reliably predict the occurrence of HF in middle-aged and elderly individuals with periodontitis. Dekker et al. [39] screened salivary biomarkers for HF and revealed good agreement with serum biomarkers. More importantly, there is a strong correlation between serum salivary CRP and HF, indicating that oral inflammation efficiently predicts the deterioration in heart health [39].

Pathological cardiomyocyte hypertrophy is an important cause of HF. *P. gingivalis*, a pathogen of periodontitis, was reported to induce hypertrophy and apoptosis of H9c2 myocardial cells via the p38, extracellular signal-regulated kinase, phosphoinositide 3-kinase and c-Jun N-terminal kinase signaling pathways [40]. Proteinases, such as calcineurin and mitogen-activated protein kinase, also participate in H9c2 hypertrophy and apoptosis induced by *P. gingivalis*. Furthermore, increased expression of nicotinamide adenine dinucleotide phosphate hydrogen oxidase 4 was observed in *P. gingivalis*-induced cardiac hypertrophy [41], indicating that oxidative stress may serve as a bridge between periodontitis and HF.

The systemic inflammation caused by periodontitis can also be attributed to HF. Experimental periodontitis rats established by ligation showed significant increases in CRP and IL-1β levels with multiplied quantities of β-myosin heavy chain-positive cardiomyocytes in the left ventricular tissue [42]. Moreover, high levels of IL-6 are associated with HF.

## 6. The Association Between Periodontitis and Cardiomyopathy

Cardiomyopathy encompasses a group of diseases characterized by abnormal cardiac structure and function, excluding other identifiable causes of myocardial abnormalities. Research conducted by Shimazaki et al. [43] reported a correlation between periodontitis and left ventricular hypertrophy (LVH). Specifically, deeper periodontal pockets were associated with an increased risk of LVH, whereas greater clinical attachment loss was associated with a higher risk of LVH in multifactorial analyses [43]. Subsequently, Franek et al. [44] confirmed that patients with periodontitis exhibited more pronounced LVH than those without periodontal disease. These clinical observations strongly suggest a link between periodontitis and LVH.

Lee et al. [40] conducted cellular experiments and discovered that *P. gingivalis* can induce cardiomyocyte hypertrophy by activating various signaling pathways. Specifically, they explored the roles of mitogen-activated protein kinase and calcineurin in this process. Additionally, the pathogen triggers cellular hypertrophy and matrix metalloproteinase-9 activity in H9c2 cardiomyocytes via distinct signaling mechanisms [40]. Other researchers found that *P. gingivalis* exacerbates pathological myocardial hypertrophy in experimental animals by inducing oxidative stress [41, 45]. Another periodontal pathogen, *A. actinomycetemcomitans*, has also been shown to worsen pressure overload-induced myocardial hypertrophy [46]. These studies highlight the potential impact of periodontal pathogens on cardiac health. In a separate study by Köse et al. [42], periodontitis led to a systemic inflammatory state that triggered oxidative stress in the coronary microvascular endothelium. Oxidative stress, characterized by increased production of reactive oxygen species, contributes to pathological myocardial hypertrophy.

Collectively, these findings highlight the need for further research on the intricate relationship between periodontitis and cardiomyopathy. The potential impact of periodontal pathogens on cardiac health remains an important research topic.

## 7. The Association Between Periodontitis and Valvular Heart Disease (VHD)

The association between VHD and periodontal health has attracted considerable attention. A prospective observational case–control study involving 30 patients with VHD and 30 controls revealed significant differences in the bacterial plaque index and probing depth between the two groups, with patients in the VHD group exhibiting higher scores [47]. Furthermore, Silvestre et al. [47] conducted a retrospective cohort study and found that the cumulative incidence of VHD was significantly higher in the periodontitis group. Specifically, the incidence of VHD was 6.44 per 1000 person-years in the periodontitis group, whereas it was 4.65 per 1000 person-years in the nonperiodontitis group [48]. The relative hazard ratio for developing VHD in the nonperiodontitis group was 1.39, indicating a higher risk associated with periodontitis [48]. Intensive treatment of periodontitis significantly reduced the risk of VHD [48]. Although evidence linking periodontitis and VHD remains limited, these findings are intriguing and suggest a potential association between the two conditions. Further studies are required to fully understand this relationship and its clinical implications.

Disruption of symbiotic relationships within the oral microbiome can lead to the migration of harmful microorganisms to normal or already compromised cardiac valves, thereby increasing the risk of permanent damage. Additionally, structural changes in the heart following the implantation of artificial heart valves or pacemakers create rough surfaces that facilitate the adherence of bacteria to the cardiac valves or endocardium. Multiple studies have examined the DNA extracted from heart valve specimens obtained during valve replacement surgery. Researchers have collected specimens from degenerative aortic valves, mitral valves, and aortic aneurysm walls and compared them with those from periodontal pathogens [49]. *Streptococcus mutans* was the most frequently isolated species, whereas the detection frequency of periodontitis-associated pathogens such as *A. actinomycetemcomitans*, *P. gingivalis*, and *Prevotella intermedia* was lower, with varying results across studies [49]. These discrepancies may be attributed to limitations related to the sample size and differences in the types of pathogens examined.

Periodontitis and VHD share numerous risk factors including aging, sex, smoking, alcohol consumption, and diabetes. Both conditions are theoretically associated with chronic inflammatory processes. Periodontitis triggers local inflammation leading to alveolar bone resorption and systemic inflammation. Clinical and animal studies have suggested the presence of active inflammation in VHD, which drives cardiovascular calcification [50]. Periodontal treatment reduces levels of both oral and systemic inflammatory markers in patients with diabetes. Therefore, managing periodontitis may be a promising approach to mitigate the risk of VHD. Although large-scale epidemiological studies are lacking, the available data underscore the need for further studies. Future research should aim to unravel the complex interactions between periodontal health and valvular function, ultimately paving the way for targeted interventions.

## 8. Periodontitis and Central Trained Immunity (TRIM)

TRIM refers to a nonspecific, epigenetically encoded memory within the innate immune system that enhances responsiveness upon secondary stimulation [51–53]. While initially protective against pathogens, TRIM may become maladaptive under chronic inflammatory conditions, such as periodontitis [51–53].

In periodontitis, microbial products and proinflammatory cytokines can disseminate systemically and activate hematopoietic stem and progenitor cells (HSPCs) in the bone marrow via pattern recognition receptors [53, 54]. This activation triggers epigenetic and metabolic reprograming, leading to a sustained myeloid-biased differentiation program known as central TRIM memory [51–53]. The resulting hyper-inflammatory neutrophils and monocytes release excessive IL-1β, reactive oxygen species, and neutrophil extracellular traps upon secondary stimuli, amplifying both local and systemic inflammation [51–53]. Li et al. [55] demonstrated that periodontitis-induced TRIM persists beyond inflammation resolution and can be transferred via bone marrow transplantation. This reprograming exacerbates inflammatory arthritis, and RA can, in turn, reinforce HSPC training, highlighting a bidirectional axis of inflammatory comorbidity. Similarly, Wang et al. [56] reported that *DNMT3A*-mutated clonal hematopoiesis (CHIP) induces a TRIM-like phenotype, characterized by chronic myeloid skewing, heightened cytokine production, and enhanced osteoclastogenesis. CHIP potentiates periodontitis, RA, and atherosclerosis, in part through paracrine activation of surrounding immune cells, forming a feed-forward loop of systemic inflammation. Moreover, *P. gingivalis* can induce trained immunity-like changes in monocytes and gingival fibroblasts, including increased IL-6 or TNF-α production, glycolytic activation, and H3K4me1 modification [57, 58]. In vivo, *P. gingivalis* expands osteoclast precursors and promotes bone loss, further implicating the bacteria in local and systemic immune reprograming [59].

Although trained immunity may be protective in acute infection, its chronic activation in periodontitis likely contributes to systemic inflammatory pathologies [53]. IL-1 signaling is a key driver of TRIM, and its inhibition via canakinumab in the CANTOS trial reduced cardiovascular and arthritic events, likely by suppressing bone marrow-driven inflammation [60]. Epigenetic and metabolic modulators, such as DNMT, HDAC, and mTOR inhibitors, represent additional therapeutic avenues [53, 56], although their clinical application remains limited by specificity and delivery challenges.

In summary, periodontitis-induced TRIM represents a unifying mechanism linking periodontal inflammation to comorbidities such as RA, CHIP, CVD, and metabolic syndrome. Therapeutically targeting this axis may offer novel strategies to mitigate both local and systemic disease burdens.

## 9. The Impact of Periodontal Therapies on CVDs

Periodontal therapy targets risk factors associated with the disease and is typically structured into the four stages, Ⅰ, Ⅱ, Ⅲ, and Ⅳ. All stages share essential treatment measures such as oral hygiene instruction patient education on oral health, and motivation at every visit to ensure effective oral hygiene habits; dental prophylaxis and rigorous biofilm control through dental care and professional supra- and sub-gingival debridement; management of other behavioral risk factors and systemic diseases when present (such as smoking, diabetes); surgical correction of bone defects and multidisciplinary intervention therapies, control of secondary occlusal trauma through orthodontic treatment; and rehabilitation missing teeth or edentulous area via prosthodontic and implant treatments. Periodontal therapy requires lifelong management, with clinicians regularly monitoring patients to encourage consistent dental visits [61, 62].

Given the association between periodontitis and CVDs, it is important to analyze the impact of periodontal therapies on CVDs, particularly on primary and secondary interventions [63, 64]. In a cross-sectional study conducted using the Scottish Health Survey, de Oliveira et al. [63] linked data from 11,869 individuals to a database of hospital admissions and death records from 1995 to 2003, with follow-up until 2007. They reported evidence of an association between oral hygiene habits (toothbrushing) and CVD events. Specifically, participants who brushed less than once a day had the highest incidence of CVD events (HR = 1.7, 95% CI = 1.3–2.3) compared to those who brushed twice a day, indicating that oral hygiene routines may reduce the incidence of CVD [63]. In a prospective population-based study including 247,696 participants without a history of CVD, conducted between 2002 and 2003, Park et al. [64] found that poor oral heath, characterized by an increased number of dental caries, presence of periodontitis, and elevated rates of teeth loss, is associated with a high risk of future major CVD events, including cardiovascular death, acute myocardial infarction, HF, and stroke. Notably, the hazard ratio for the incidence of CVD events was decreased in individuals who increased their daily toothbrushing frequency by one episode (HR = 0.91, 95% CI = 0.89–0.93), with regular professional cleaning further reducing this risk (HR = 0.86, 95% CI = 0.82–0.90) [64]. In addition, a cohort study demonstrated that patients with periodontitis who exhibited a poor response to periodontal treatment had a higher incidence of CVDs (incidence rate [IR] = 1.28, 95% CI = 1.07, 1.53) than that of good responders, suggesting that successful periodontal therapy may reduce the risk of CVDs [65].

A recent prospective cohort study on secondary prevention of CVDs found no significant associations between periodontitis variables measured at Ramfjord teeth and major adverse cardiovascular event (MACE) incidence. However, each additional missing tooth was associated with an increased hazard of MACE (HR = 1.03, 95% CI = 1.01, 1.05) [66]. Currently, studies on the impact of periodontal therapy studies on secondary prevention of CVDs remain lacking, The feasibility of conductingadequately powered clinical studies to better clarify the impact of periodontal treatment on secondary prevention of CVDs at a population level remains uncertain owing several limitations, including recruiting a sufficient number of participants, ensuring adequate follow-up duration, and controlling confounding risk factors such as diabetes, obesity, or smoking.

## 10. Discussion

The association between periodontitis and CVDs, including CHD, HTN, AF, and VHD, has received extensive research attention (Tables 1, 2). An epidemiological study confirmed that periodontitis plays a significant role in CVD and may be an independent cardiovascular risk factor [67]. Despite these significant associations, the exact causal relationship between periodontitis and CVD remains unclear. Therefore, multicentre, high-quality studies are urgently needed to assess the prevalence and distribution of periodontitis in patients with CVD, especially among high-risk populations. Although studies have revealed that treating periodontitis may have positive effects on CHD and HTN, its effect on reducing CVD events such as LVH and VHD still needs clarification [24, 68]. To ascertain the benefits of periodontitis treatment in lowering CVD risk, standardized definitions of periodontitis and randomized clinical trials of periodontal interventions are needed. Additionally, more extensive and long-term studies should explore its impact on patients' cardiovascular health to reduce the incidence and mortality rates.

Current research has emphasized the potential importance of periodontitis as a risk factor for CVD and has revealed various mechanisms of interaction between the two conditions. These mechanisms include oxidative stress, immune-inflammatory responses and dysbiosis of the oral microbiota. Periodontitis may directly or indirectly induce systemic inflammation and oxidative stress by altering the circulation of oral microbiota, thereby affecting the occurrence and development of CVD. However, further research is needed to confirm and elucidate the key role of periodontal microbial imbalance in CVD and the specific mechanisms underlying the interaction between the oral microbiota and the host. Exploring the interplay between cardiovascular and periodontitis risk exposures and host responses will help us better understand the complex relationship between periodontitis and CVD.

The interaction between periodontitis and CVD has garnered scientific interest, supported by in vitro, preclinical and clinical studies [67]. However, interventional trials are yet to provide conclusive evidence. Future research should explore the impact of expanded periodontal therapy on systemic inflammation and endothelial function markers, as well as how these changes affect cardiovascular events and risks. The strength of the association between periodontitis and CVD may vary depending on the study populations. Given the higher prevalence of periodontitis in elderly participants, future investigations should consider aging as an important factor in the association between periodontitis and CVD.

Further research in this field not only has the potential to bridge the gap between oral diseases and CVD, providing more evidence for integrated prevention and control, but may also offer new perspectives for the prevention and treatment of these diseases. Additionally, emerging imaging techniques such as echocardiography and coronary computed tomography angiography should be considered to detect relevant cardiovascular disease markers and assess their relationship with periodontitis. Furthermore, novel diagnostic and monitoring methods using validated alternative biomarkers could provide a fresh perspective for evaluating periodontal treatment outcomes independent of any association with other systemic diseases.

In summary, the association between periodontitis and CVD warrants further exploration. By strengthening prevention strategies and management of periodontitis, we have the potential to improve both oral and overall health, enhance quality of life, and possibly prevent or ameliorate cardiovascular conditions, such as HTN, LVH, VHD, and CHD. Through ongoing research, we may gain new perspectives and approaches for the prevention and treatment of CVD.

## Figures and Tables

**Table 1:** The impact of periodontitis on CVDs.

Study (author, year)	Study design	Population/timeframe	Sample size (*N*)	Key estimates (95% CI)	Effect on CVDs
Emingil et al. 2000 [7]	Case–control study	Acute myocardial infarction and chronic coronary heart disease	120	Model chi square = 31,175, R2 = 76.81%.	Yes
Sumayin Ngamdu et al. 2022 [8]	Cross-sectional study	Periodontal disease (from 2013 to 2014)	2830	OR = 3.59, 95% CI = 1.12–11.54	Yes
Bilgin Çetin et al. 2020 [9]	Cross-sectional study	Coronary artery disease (+) and coronary artery disease (−)	309	OR = 2.48, 95% CI = 1.24–4.95; OR = 2.01, 95% CI = 1.14–5.17	Yes
Gao et al. 2021 [10]	Cohort study	CHD and periodontitis (2014 at baseline and 2019 at follow-up)	4591	RR = 1.37; 95% CI = 0.96–1.95	Yes
Bahekar et al. 2007 [11]	Meta-analysis	CAD and periodontal disease	86,092	RR = 1.14, 95% CI = 1.074–1.213	Yes
Nepomuceno et al. 2017 [12]	Meta-analysis	With and without chronic periodontitis	19 studies	/	PD is significantly associated with reduction of HDL and elevation of LDL and triglyceride concentrations
Atarbashi-Moghadam et al. 2018 [13]	Cross-sectional study	CAD and moderate to severe periodontitis	23	*P.g, A.a*, and *C.r* of subgingival plaques: 43.47%, 43.47%, and 78.26%; atherosclerotic plaques: 13.04%, 17.39%, and 8.69%	The presence of periopathogens in atherosclerotic plaques of patients with chronic periodontitis
Ogawa et al. 1998 [20]	Cross-sectional study	Male Japanese factory workers	About 2000	/	There was significant relationship between periodontal disease and the prevalence of hypertension
Muñoz Aguilera et al. 2020 [21]	Meta-analysis	With and without chronic periodontitis	40 studies	Moderate–severe PD (OR = 1.22; 95% CI = 1.10–1.35); severe PD (OR = 1.49; 95% CI: 1.09–2.05)	Yes
Lu et al. 2024 [22]	Meta-analysis	Periodontitis to atherosclerotic cardiovascular disease in people with metabolic syndrome components components	19 studies	/	Yes
Desvarieux et al. 2010 [23]	Cross-sectional study	Dentate men and women with no history of stroke or myocardial infarction	653	Etiologic bacterial burden was positively associated with hypertension (OR: 3.05; 95% CI: 1.60–5.82)	Yes
Struppek et al. 2021 [29]	Cross-sectional study	Patients without AF	5634	OR = 1.22, 95% CI = 1.1–1.35	Dental plaque index was associated with AF
Zhang et al. 2023 [30]	Systematic reviews and meta-analysis	Periodontitis and other oral inflammatory diseases (from establishment of each database to 2022.11)	4,328,355	/	Regular and moderate oral hygiene, frequent tooth brushing, and prevention of PD and other oral inflammatory diseases could reduce the occurrence of AF
Miyauchi et al. [31]	Cohort study	AF (17.1 ± 14.5 months follow-up)	596	Paroxysmal AF: HR = 1.569, 95% CI = 1.010–2.427; nonparoxysmal AF: HR = 1.909, 95% CI = 1.213–3.005	Yes
Sen et al. 2021 [32]	Cohort study	Patients without AF (17 years follow-up)	5958	HR = 1.31, 95% CI = 1.06–1.62	Only severe PD increased the risk of AF
Miyauchi et al. 2023 [33]	Prospective study	AF	76	*R* = 0.57*⁣*^*∗*^; β = 0.016*⁣*^*∗*^	Histologically revealed the association of periodontitis with atrial fibrosis
Yan et al. 2022 [37]	Cross-sectional study	No/mild periodontitis and moderate/severe periodontitis	13,202	OR = 5.72, 95% CI = 3.76–8.72	Yes
Huh et al. 2023 [38]	Cohort study	Type 2 diabetes (10 years follow-up)	173,927	Brushed teeth ≥ 2 times/day (HR = 0.90, 95% CI = 0.82–0.98); professional dental cleaning ≥ 1 time/year (HR = 0.93, 95% CI = 0.87–0.99)	Among patients with type 2 diabetes, dental diseases and oral hygiene care are important determinants of HF development
Dekker et al. 2017 [39]	Cross-sectional study	Patients with HF	75	A moderate-to-strong correlation between serum and salivary CRP (*r*_s_*⁣*^*∗*^ = 0.594, *p* < 0.001)	Salivary CRP and IL-6 concentrations correlated with serum measures in patients with HF
Shimazaki et al. 2004 [43]	Cross-sectional study	Residents of Hisayama town, Fukuoka, Japan	1111	OR = 1.6; 95% CI = 1.01 to 2.50 OR = 1.7; 95% CI = 1.07 to 2.67	Yes
Franek et al. 2005 [44]	Cross-sectional study	With advanced and patients without or with moderate periodontal lesions	99	/	Advanced PD in patients after kidney transplantation is associated with LVH
Silvestre et al. 2017 [47]	Prospective, observational case–control study	With heart valve disease and controls	60	/	60 percent of the patients with valve disease presented periodontitis
Sia et al. 2021 [48]	Cohort study	With and without periodontitis	262,552	HR = 0.68 95% CI = 0.60–0.77	Yes

*⁣* ^*∗*^Statistically significant (*p* < 0.001).

**Table 2:** The impact of periodontal therapies on CVDs.

Study (author, year)	Study design	Topic	Sample size (*N*)	Outcome
de Oliveira et al. 2010 [63]	Cross-sectional study	Primary prevention	11,869 (from 1995 to 2003 and follow-up until 2007)	Brushing less than once a day had the highest incidence of CVD events (HR = 1.7, 95% CI = 1.3, 2.3)
Park et al. 2019 [64]	Prospective population-based study	Primary prevention	247,696 (between 2002 and 2003)	Incidence of CVD events was reduced more in the group of people who conducted one additional toothbrushing episode per day (HR = 0.91, 95% CI = 0.89, 0.93) and regular professional cleaning reduced the risk even further (HR = 0.86, 95% CI = 0.82, 0.90)
Holmlund et al. 2017 [65]	Cohort study	Primary prevention	8999 (between 1979 and 2012)	Poor response to periodontal treatment exhibited an increased incidence of CVDs (incidence rate (IR) = 1.28, 95% CI = 1.07, 1.53)
Hamaya et al. 2023 [66]	Cohort study	Secondary prevention	888 (between 2012 and 2015)	An additional missing tooth was associated with a higher hazard of MACE (HR = 1.03, 95% CI = 1.01, 1.05)
Czesnikiewicz-Guzik et al. 2019 [24]	Randomized controlled trial	Hypertensive patients with moderate/severe periodontitis	101 (between 2009 and 2015)	A substantial reduction in mean SBP in IPT compared to the CPT (mean difference of −11.1 mmHg; 95% CI 6.5–15.8)

## Data Availability

Data sharing is not applicable to this article as no datasets were generated or analyzed during the current study.

## Nomenclature

CVD: Cardiovascular disease
HF: Heart failure
CHD: Coronary heart disease
AF: Atrial fibrillation
IL: Interleukin
RGS1: G protein signaling
CRP: C-reactive protein
HTN: Hypertension
SBP: Systolic blood pressure
DBP: Diastolic blood pressure
NO: Nitric oxide
LVH: Left ventricular hypertrophy
VHD: Valvular heart disease.
